# Exosomes from Human Omental Adipose-Derived Mesenchymal Stem Cells Secreted into Ascites Promote Peritoneal Metastasis of Epithelial Ovarian Cancer

**DOI:** 10.3390/cells11213392

**Published:** 2022-10-27

**Authors:** Qingxi Qu, Linghong Liu, Yuqian Cui, Yu Chen, Yu Wang, Yaodu Wang

**Affiliations:** 1 Department of Obstetrics and Gynecology, Qilu Hospital of Shandong University, Jinan 250012, China; 2 Research Center of Stem Cell and Regenerative Medicine, Shandong University, Jinan 250012, China; 3 Laboratory of Cryomedicine, Qilu Hospital of Shandong University, Jinan 250012, China; 4 Department of Radiation Oncology, Qilu Hospital of Shandong University, Jinan 250012, China

**Keywords:** exosomes, mesenchymal stem cells, epithelial ovarian cancer, peritoneal metastasis, human omentum, ascites, MSC: Mesenchymal stem cell, EOC: Epithelial ovarian cancer, ADSCs: Adipose-derived mesenchymal stem cells, HO-ADSC: Human omental Adipose-derived mesenchymal stem cell, HO-CM: Human omental tissue conditioned medium, exosomes secreted by HO-ADSCs: HO-ADSC exosomes, IHC: Immunohistochemistry, qRT-PCR: Quantitative real-time PCR, SBT: Subcutaneous tumorigenesis, PMX: Peritoneal metastatic xenograft, TEM: Transmission electron microscopy, HE: Hematoxylin and eosin

## Abstract

Epithelial ovarian cancer (EOC) patients frequently develop peritoneal metastasis, especially in the human omentum. However, the mechanism underlying this propensity remains unknown. A previous study found that human omental adipose-derived mesenchymal stem cells are potentially involved in ovarian cancer growth and metastasis, but the results were inconsistent and even contradictory. In addition, the underlying mechanisms of visceral adipose metastasis remain poorly understood. Here, our goal is to clarify the role and mechanism of human omental adipose-derived mesenchymal stem cells (HO-ADSCs) in EOC cancer growth and metastasis. We first found that human omental tissue conditioned medium (HO-CM) enhances EOC cell function. Subsequent coculture studies indicated that HO-ADSCs increase the growth, migratory and invasive capabilities of ovarian cancer cells. Then, we demonstrated that exosomes secreted by HO-ADSCs (HO-ADSC exosomes) enhanced ovarian cancer cell function, and further mechanistic studies showed that the FOXM1, Cyclin F, KIF20A, and MAPK signaling pathways were involved in this process. In addition, subcutaneous tumorigenesis and peritoneal metastatic xenograft experiments provided evidence that HO-ADSC exosomes promote ovarian cancer growth and metastasis in vivo. Finally, our clinical studies provided evidence that ascites from ovarian cancer patients enhance EOC cell line proliferation, migration, and invasion in vitro. The present study indicated that HO-ADSC exosomes are secreted into ascites and exert a tumor-promoting effect on EOC growth and metastasis, providing a new perspective and method to develop future novel therapeutic strategies for the treatment of ovarian cancer.

## 1. Introduction

Ovarian malignancies can occur at any age and have a wide range of histological types, especially epithelial ovarian cancer (EOC). EOC is the most common cause of cancer-related death among all gynecological malignancies. EOC patients often present at an advanced stage with peritoneal dissemination and extensive metastases and are commonly complicated with ascites [1]. More than 60% of cases are detected after cancer has spread into the abdomen. Previous studies have emphasized that the omentum is one of the most common regions of metastasis in EOC, and 80% of serous ovarian cancers are commonly found at the site of the omentum [2]. However, the molecular mechanisms underlying this lipophilic metastatic characteristic remain poorly understood. In view of the rapid progression, early metastasis, and low survival rate in EOC, it is of great significance to clarify the molecular mechanisms of invasion and metastasis and identify new targets in the treatment of ovarian cancer.

The omentum, a large fat pad that serves as a rich source of adipocytes and adipose-derived mesenchymal stem cells (ADSCs), is historically known as the “policeman of the abdomen” [3]. It is widely accepted that the omentum is involved in the immune response and fluid exchange of the human abdominal cavity [4]. In addition, it is becoming increasingly apparent that the omentum is directly involved in the development of a series of cancers, including ovarian cancer, gastric cancer, and pancreatic cancer [5,6,7]. Previous research has suggested that adipocytes provide energy for the rapid growth of ovarian cancer and enhance tumor cell metastasis [2]. Mesenchymal stem cells (MSCs) are involved in the development of malignant tumors [8]. The omentum is the most common metastatic site of ovarian cancer, and ADSCs are in situ MSCs. However, it seems that the specific relationships between ADSCs and ovarian cancer cells remain unknown [9,10]. Previous studies have reported that ADSCs can promote tumor progression and ovarian cancer metastasis by regulating several regulatory factors, such as MMPs, STAT3, and TMSB4X [11,12,13]. For example, the study reported by Chu Y et al. showed that human omental adipose-derived MSCs (HO-ADSCs) enhance autophagy in ovarian carcinoma cells by activating the STAT3 signaling pathway [12]. Evidence has shown that ADSCs exhibit an inhibitory influence on ovarian cancer aggressiveness by targeting different paracrine molecules [14,15]. Accordingly, it is essential to research the mechanisms underlying the contradictory roles associated with HO-ADSCs in EOC tumorigenesis and progression.

Exosomes are a special class of extracellular enclosed vesicles with small membranes secreted by various cell types [16]. These exosomes can transfer proteins, lipids, and nucleic acids into different recipient cancer cells, thus affecting their malignant biological behavior [17]. Xiaoli Rong revealed that human bone marrow MSC-derived exosomes (MSC-exosomes) ameliorate liver fibrosis via inhibition of hepatic stellate cell activation [18]. Olga Kersy showed that omental tissue-derived exosomes enhanced gastric cancer growth and invasion [19]. However, analysis concerning the involvement of exosomes secreted by ADSCs (ADSCs-exosomes) in the tumorigenesis and metastasis of ovarian cancer is limited. To the best of our knowledge, there are no reports regarding the impact of ADSC-exosomes on the interaction between the omentum and ovarian cancer cells to date.

In this paper, we explored the functional role of HO-ADSCs and their underlying mechanism in EOC growth and metastasis in vivo and in vitro. We demonstrated that HO-ADSCs promote EOC growth and metastasis through the exosome-mediated FOXM1 signaling pathway. We inferred that HO-ADSC exosomes may be secreted into ascites and are involved in ovarian cancer growth and metastasis, and these properties have potential clinical applications.

## 2. Materials and Methods

### 2.1. Omental Adipose Tissue and Ascites Specimens

Written informed consent was acquired from all participants pathologically diagnosed with ovarian cancer before inclusion in this study. None of the patients had received preoperative chemotherapy, radiotherapy, or other therapies prior to the surgical treatment. The protocol of this study was approved by the Ethics Committee of Qilu Hospital of Shandong University (approval number KYLL-202111-134). Accordingly, we followed the ethical principles for medical research involving human subjects as outlined in the Helsinki Declaration. The omental adipose tissue samples and ascites specimens were freshly collected from four middle-aged women aged who underwent surgical treatment for the first time and were diagnosed with advanced ovarian cancer with omentum metastasis at the Department of Obstetrics and Gynaecology, Qilu Hospital of Shandong University. Omental adipose tissue was directly processed for primary ADSC isolation, and the fresh ascites were centrifuged to remove cell components for further cell function studies.

### 2.2. ADSC Culture, Isolation, and Identification

ADSCs were isolated from omental adipose tissue samples by the explant method based on a protocol described previously [20]. The tissue samples from the patients were washed, minced, further digested with collagenase, cleaned, centrifuged, resuspended, and cultured in DMEM (Gibco BRL, Gaithersburg, MD, USA) supplemented with 20% fetal bovine serum (FBS, Gibco BRL, Gaithersburg, MD, USA. The isolated cells were cultured in a common incubator at 37 °C with 5% CO_2_ and 95% air, and cells from passages 3 to 5 were used in this study. Stem cell multilineage differentiation assays were used to assess the differentiation of HO-ADSCs by Oli Red O, Alizarin Red, and Alcian Blue staining. Fluorescein isothiocyanate-conjugated CD73, CD90, CD105, CD11, CD19, CD34, CD45, and HLA-DR antibodies (Proteintech, Wuhan, China)were employed to assess the phenotypic purity of HO-ADSCs by flow cytometry.

### 2.3. Coculture of Ovarian Cancer Cells and HO-ADSCs

EOC cell lines (SKOV3, Hey, A2780, and HO8910) were maintained in DMEM or McCoy’s 5A medium containing 10% FBS as previously described [21]. EOC cell lines were cocultured with HO-ADSCs using Transwell chambers (0.4-μm pore size, Corning, USA) as previously described [22]. HO-ADSCs and EOC cell lines were suspended at a density of 4 × 106 cells/mL and seeded together in plates or inserts with a 6-well transwell chamber. Subsequently, the medium was replaced half a day to ensure sufficient nutrients and HO-ADSCs were cocultured with EOC cell lines for 24 h. The single-cultured EOC cell lines were used as the control. After incubation for 24 h, ovarian cancer cells were collected and subjected to functional analysis and mechanistic studies.

### 2.4. EdU Staining Assay

Cell proliferation was measured using an EdU Cell Proliferation Kit (RiboBio, Guangzhou, China) according to the protocol we previously reported [23]. Briefly, ovarian cancer cells were seeded into 24-well plates and treated with proper interventions for 24 h. Afterward, the cells were incubated with EdU reagents for 2 h and then fixed with paraformaldehyde for 10 min. Finally, the nuclei were stained with Hoechst 3342, and the EdU-positive cells in different groups were counted under a microscope in 5 random fields. Each of the above experiments was performed at least thrice.

### 2.5. Transwell Migration Assay

Cell migration assays were performed using a Transwell chamber (8.0 μm pore size, Corning, Glendale, AZ, USA) according to the manufacturer’s instructions. Briefly, EOC cell lines were placed into the upper chamber with an FBS-free medium, and the bottom chamber routinely contained a complete medium. After incubation at 37 °C for 24 h, cells in the upper chamber were fixed with paraformaldehyde and stained with crystal violet. Then, the cells that did not migrate were carefully removed from the upper surface of the chamber, and the cells present on the transmembrane located on the underside of the chamber were processed for further analysis. Finally, the stained cells were randomly imaged and manually counted using an inverted microscope. Each of the above experiments was performed at least thrice.

### 2.6. Matrigel Invasion Assay

Transwell chambers (8 µm pore size, Corning, Glendale, AZ, USA) coated with Matrigel were utilized to measure invasive cell ability, as previously reported, with some modifications. In brief, Matrigel was first added to the upper chamber (BD Biosciences, San Diego, CA, USA). Then, EOC cell lines were seeded in the inserts with serum-free medium, whereas complete medium was added into the lower chamber. After 24 h of incubation in an incubator, human EOC cell lines in the upper chamber were fixed with paraformaldehyde and stained with crystal violet, and then the cells that did not exhibit invasive properties were carefully removed from the upper surface of the chamber using a cotton swab. Subsequently, the cells that had passed through the membrane were randomly counted using an inverted microscope. Each of the above experiments was performed at least thrice.

### 2.7. ADSC-Exosome Extraction, Characterization, Labeling, and Uptake

To extract exosomes from HO-ADSC supernatants, cells were initially incubated with an exosome-free medium for 48 h. Then, the ADSC exosomes were isolated and purified from the cell supernatants by gradient ultracentrifugation according to a standard protocol. Briefly, the culture medium supernatant of HO-ADSCs was collected and centrifuged at 1500× *g* for 10 min and 10,000× *g* for 30 min at 4 °C to remove the cells and debris. The processed supernatant was further centrifuged at 120,000× *g* for 90 min at 4 °C to obtain the final exosomal pellet. The exosome protein concentration was quantified by employing a BCA Protein Assay Kit (Beyotime, China), and Western blotting was used to identify exosome-specific markers (CD63, TSG101, and HSP70). The morphology and size of exosomes were observed using a transmission electron microscope (JEOL, AICHI KEN, Japan). To detect the cellular uptake and internalization of exosomes, exosomes were labeled with PKH67 dye for 15 min and then incubated with EOC cell lines for 12 h at 37 °C. After incubation, cell nuclei were stained with DAPI for 5 min and subsequently visualized using a fluorescence microscope. The exosome pellets were resuspended in PBS and stored in a −80 °C refrigerator.

### 2.8. RNA Sequencing and Analysis

For RNA sequencing, total RNA from three paired HEY cells with or without HO-ADSC-exosome treatment was extracted using TRIzol Reagent (Thermo Fisher Scientific, Waltham, MA, USA). After using the Agilent 2100 RNA nano 6000 assay kit (Agilent, San Diego, CA, USA) to analyze the quality and concentration of RNA samples, the mRNA was enriched by magnetic beads attached to oligos. Then, the first strand and the second strand of cDNA were synthesized and purified using a QIAquick PCR Purification Kit (Qiagen, Frankfurt, Germany). The purified double-stranded cDNA was amplified, and the cyclization product was qualified. The qualified cyclized products were attached to DNB, and the constructed DNB was loaded into an MGI platform sequencer for computer sequencing. The PE150 sequencing strategy was employed. The information analysis process mainly included sequencing data quality control, data comparison analysis, and in-depth transcriptome analysis. The annotated differentially expressed genes were processed based on Gene ontology (GO) and Kyoto Encyclopedia of Genes and Genomes (KEGG) analysis via KOBAS software. Whole transcriptome sequencing depth analysis and personalized analysis were performed at Ruizhi Bio Ltd. (Jinan, China).

### 2.9. In Vivo Xenograft Experiments

All nude mice (female BALB/c nude mice, 4 weeks old) were purchased from Nanjing Model Animal Research Center and maintained under special pathogen-free conditions with access to water and food. Twenty-four mice were used in the experiments to reduce the use of animal testing. We established both the subcutaneous xenograft model and peritoneal metastatic xenograft model; both of them have two subgroups, including the PBS group (n = 6 mice/group) and HO-ADSC-exosome group (n = 6 mice/group). For the subcutaneous xenograft model, the suspended EOC cell line HEY cells (1 × 10^5^ cells in 100 µL/mouse) were subcutaneously injected into the right shoulder of mice (5 weeks old, 18–22 g). On Day 7, following cell injection, the xenograft tumors were injected with HO-ADSC exosomes or an equal volume of PBS for 7 days. For the peritoneal metastatic xenograft model, nude mice were injected with a similar number of EOC cells into the abdominal cavities, followed by intraperitoneal injection with HO-ADSC exosomes or PBS, as mentioned above. After the administration of HO-ADSC exosomes for 28 days, the mice were sacrificed via cervical dislocation, and the tumor tissues were collected. Tumors were weighed and photographed. The volumes of the tumors were calculated, and the number of metastatic nodules was counted. Thereafter, tumor tissues were stripped for further Western blotting or immunohistochemistry assays. Animal experiments were approved by the Ethical Committee for Animal Research (Permit number DWLL-2021-019) and were performed in strict compliance with the Guide for the Care and Use Laboratory Animals of Qilu Hospital of Shandong University.

### 2.10. Immunohistochemistry (IHC)

Immunohistochemistry was performed according to a previously described method [23]. Briefly, all fresh ovarian cancer samples were initially incubated with paraformaldehyde, paraffin-embedded, and sliced into sections. Subsequently, the slides were deparaffinized with xylene, hydrated with alcohol, heated in EDTA solution, and blocked with BSA. Then, the slides were incubated with primary antibodies, including Ki-67, caspase-3, MMP2, MMP9, and FOXM1, for 1 h before incubation with peroxidase-labeled anti-rabbit (or anti-mouse) secondary antibodies for 30 min. Finally, the chromogenic procedures were processed with 3, 3′-diaminobenzidine solution and stained with hematoxylin reagents. Histological images were obtained using a microscope and quantitatively analyzed with ImageJ software.

### 2.11. Western Blot Assay

Total protein was collected from EOC cell lines or xenograft tumours of nude mice using RIPA lysis buffer (Beyotime, Beijing, China), and protein concentrations were determined by using a BCA protein assay kit. The protein samples were initially separated by sodium dodecyl sulfate–polyacrylamide gel electrophoresis (Beyotime, Beijing, China) and transferred onto polyvinylidene difluoride membranes (Millipore Corp, billerica, MA, USA). Then, the membrane was blocked with nonfat milk for 2 h and incubated with specific primary antibodies overnight at 4 °C, followed by incubation with horseradish peroxidase-conjugated secondary antibodies for 2 h at 37 °C. The peroxidase signal of proteins was visualized using an enhanced chemiluminescence substrate (Millipore Corp, billerica, MA, USA), and the bands were quantified using ImageJ software. GAPDH antibody was used as a housekeeping gene for normalization.

### 2.12. Quantitative Real-Time PCR (qRT–PCR)

Total RNA was extracted from EOC cell lines or xenograft tumors of nude mice using TRIzol reagent (Invitrogen, Waltham, MA, USA) and quantified using a NanoDrop DS-11 spectrophotometer (NanoDrop, Gaithersburg, MD, USA). Total RNA was subsequently reverse transcribed into cDNA using a ReverTra Ace q-PCR RT Kit (Toyobo, Shiga, Japan) according to the manufacturer’s specifications. To detect RNA expression, qRT–PCR procedures were conducted by using an SYBR Premix Ex Taq II Kit (Toyobo, Shiga, Japan) and a BioRad CFX96 Real-Time PCR detection system (BioRad, Hercules, CA, USA). Each sample was tested in triplicate. GAPDH served as endogenous control, and relative messenger RNA expression levels were calculated using the 2−ΔΔCt method in the final analysis. The MIQE guidelines for real-time PCR were followed in this study.

### 2.13. Statistical Analysis

Data are presented as the average ± standard deviation (SD) and were analyzed using SPSS 22.0 software (SPSS, Chicago, IL, USA) and GraphPad Prism 8.0 software (GraphPad, San Diego, CA, USA). In all analyses, data differences were assessed for significance using the Student’s t test or one-way analysis of variance with the post Student–Newman–Keuls test, as appropriate. Differences were considered statistically significant at a *p* value < 0.05. Based on the consideration of the research objective and cell experimental characteristics, as well as the overall homogeneity of primary cells, the samples were freshly collected from four ovarian cancer patients. All data were obtained from three separate experiments.

## 3. Results

### 3.1. Human Omental Tissue Induces Ovarian Cancer Cell Proliferation, Migration, and Invasion

**Figure 1:**
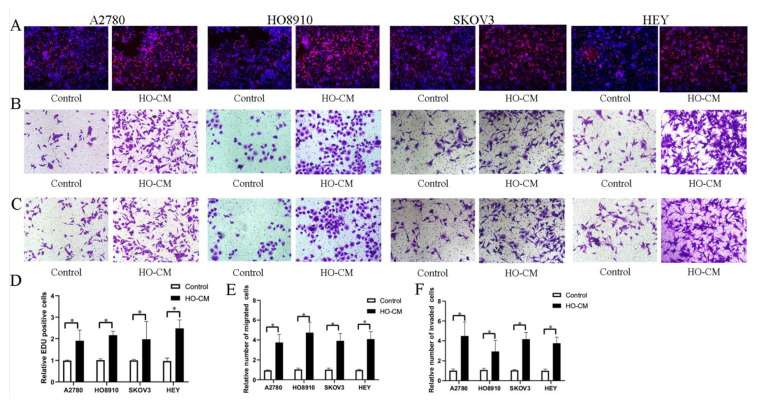
HO-CM increases human EOC cell proliferation, migration, and invasion (A2780, HO8910, SKOV3, and HEY) in vitro. (**A**). The effect of HO-CM on human EOC cell proliferation was measured by EdU assay. (**B**). The effect of HO-CM on human EOC cell line migration was measured by Transwell assay. (**C**). The effect of HO-CM on human EOC cell invasion was measured by Transwell assay. (**D**). Quantification of proliferation rates showed that the proliferative capacity of human EOC cell lines was significantly improved after HO-CM treatment. (**E**). Quantification of migrating cells showed that the migratory capacity of human EOC cell lines was significantly improved after HO-CM treatment. (**F**). Quantification of the invasion rates of human EOC cell lines showed that the invasive capacity of human EOC cell lines was significantly improved after HO-CM treatment. * *p* < 0.05, n = 3.

To study and analyze the impact of human omentum on ovarian cancer growth and metastasis, we used four human EOC cell lines (A2780, HO8910, SKOV3, and HEY) to assess the potential effects of human omental tissue conditioned medium (HO-CM) on ovarian cancer progression and metastasis. The EdU assay was utilized to evaluate cell proliferation, and the Transwell assay was utilized to determine cell invasion and migration. EdU assay results shown in Figure 1A,D demonstrated that HO-CM treatment significantly increased the percentage of proliferating cells compared with the control. The results of the migration assay showed that HO-CM enhanced migratory ability in the A2780, HO-8910, SKOV3, and HEY cell lines (Figure 1B,E). Cell invasion assays showed that HO-CM significantly increased cell mobility compared with the control (Figure 1C,F). Taken together, our results indicated that human omental tissue induced EOC cell proliferation, migration, and invasion by releasing uncharacterized factors into the culture medium and that the human omentum plays a stimulatory role in promoting ovarian cancer growth and metastasis.

### 3.2. HO-ADSCs Promote Ovarian Cancer Cell Proliferation, Migration, and Invasion In Vitro

**Figure 2:**
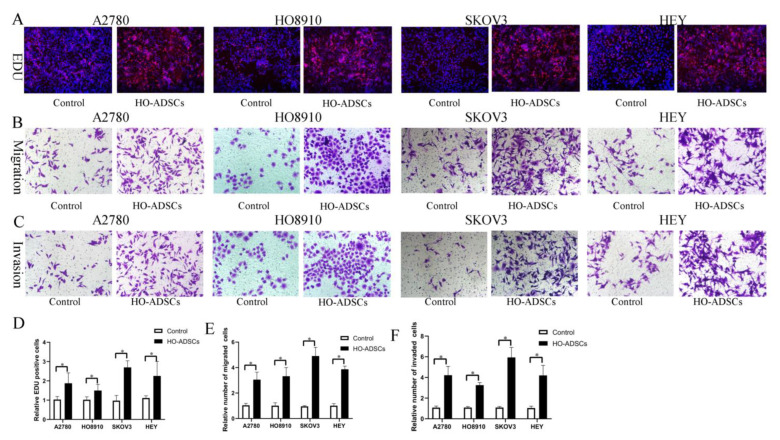
HO-ADSCs promote human EOC cell proliferation, migration, and invasion (A2780, HO8910, SKOV3, and HEY) in vitro. (**A**). The influence of HO-ADSC on human EOC cell proliferation was measured by EdU assay. (**B**). The influence of HO-ADSC on human EOC cell migration was measured by Transwell assay. (**C**). The influence of HO-ADSC on human EOC cell invasion was measured by Transwell assay. (**D**). Quantification of proliferation rates showed that the proliferative capacity of human EOC cell lines was significantly improved when cocultured with HO-ADSCs. (**E**). Quantification of the migrating cells showed that the migratory capacity of human EOC cell lines was significantly improved when cocultured with HO-ADSCs. (**F**). Quantification of the invasion rates of human EOC cell lines showed that the invasive capacity of human EOC cell lines was significantly improved when cocultured with HO-ADSCs. * *p* < 0.05, n = 3.

A previous study found that ADSCs potentially participate in ovarian cancer growth and metastasis, but the results were inconsistent and even contradictory [9,10]. In this study, we isolated ADSCs from human omental tissue (HO-ADSCs) and characterized them using a series of experiments as previously described. We determined that HO-ADSCs showed typical fibroblast-like morphology (Supplemental Figure S1A). The results of multilineage differentiation assays (Oli Red O staining, Alizarin Red staining, and Alcian Blue staining) revealed that the cultured cells had the ability to differentiate into adipogenic, osteogenic, and chondrogenic cells (Supplemental Figure S1B–D). In addition, ADSCs were highly positive for MSC surface markers, including CD73, CD90, and CD105, but negative for CD11, CD19, CD34, CD45, and HLA-DR, as demonstrated by flow cytometry analysis (Supplemental Figure S2A–F). These results verified that the extracted HO-ADSCs exhibited MSC traits. To test the effects of HO-ADSCs on ovarian cancer cell proliferation, migration, and invasion, EOC cell lines (A2780, HO8910, SKOV3, and HEY) were cocultured with HO-ADSCs for 3 days. In functional assays, we observed a significant increase in EOC cell line proliferation, migration, and invasion after coculture with ADSCs compared with the control (Figure 2A–F). Collectively, these data show that HO-ADSCs could increase the growth, migratory and invasive capabilities of EOC cells, potentially promoting ovarian cancer growth and metastasis.

### 3.3. Exosomes Are Involved in HO-ADSC-Mediated Promotion of Ovarian Cancer Cell Proliferation, Migration, and Invasion In Vitro

**Figure 3:**
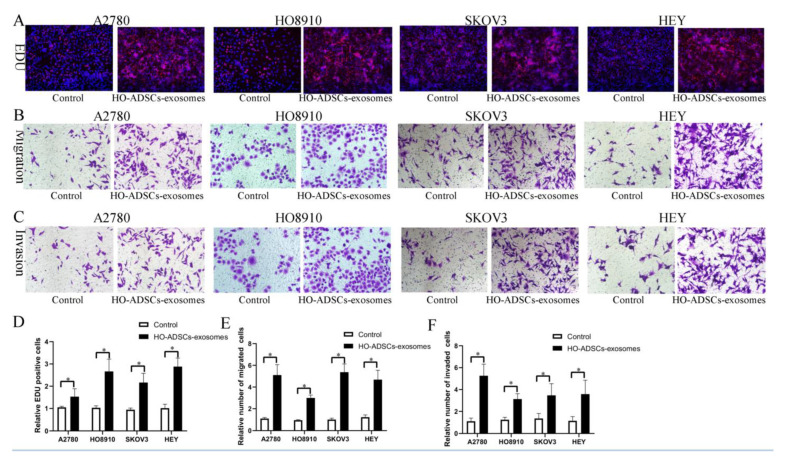
HO-ADSC exosomes promote human EOC cell proliferation, migration, and invasion (A2780, HO8910, SKOV3, and HEY) in vitro. (**A**). The influence of HO-ADSC exosomes on human EOC cell proliferation was measured by EdU assay. (**B**). The influence of HO-ADSC exosomes on human EOC cell migration was measured by Transwell assay. (**C**). The influence of HO-ADSC exosomes on human EOC cell invasion was measured by Transwell assay. (**D)**. Quantification of proliferation rates showed that the proliferative capacity of human EOC cell lines was significantly improved when exposed to HO-ADSC exosomes. (**E**). Quantification of the migrating cells showed that the migratory capacity of human EOC cell lines was significantly improved when exposed to HO-ADSC exosomes. (**F**). Quantification of the invasion rates of human EOC cell lines showed that the invasive capacity of human EOC cell lines was significantly improved when exposed to HO-ADSC exosomes. * *p* < 0.05, n = 3.

To date, accumulating evidence has indicated that exosomes represent the main method of intercellular communication. In this study, we hypothesized that exosomes might be involved in HO-ADSC-induced phenotypic and functional alterations in EOC cells. Therefore, we purified HO-ADSC exosomes from the cell-conditioned medium of HO-ADSCs and identified them by TEM and WB analysis as described. TEM and particle diameter analysis demonstrated that HO-ADSC exosomes had typical saucer-like membrane characteristics (Supplemental Figure S3A). In addition, the surface markers of exosomes detected by WB showed that the nanoparticles were positive for the known exosomal markers CD63, TSG101, and HSP70 (Supplemental Figure S3B). These data confirmed that the nanoparticles exhibited characteristics in accordance with the defined exosomes. We then examined whether HO-ADSC exosomes could be internalized into ovarian cancer cells. The results showed that PKH26-labeled exosomes could be transferred into the cytoplasm of EOC cells, as determined by an inverted fluorescence microscope (Supplemental Figure S4). To determine the effect of HO-ADSC exosomes on the biological functions of ovarian cancer cells, EdU and Transwell assays were performed using A2780, HO8910, SKOV3, and HEY cells. The EdU proliferation assay showed that HO-ADSC exosomes remarkably promoted ovarian cancer cell line proliferative activity compared with the control (Figure 3A,D). Transwell migration and invasion assays revealed that HO-ADSC exosomes significantly increased migration and invasion cell numbers compared to control cells (Figure 3B,C,E,F). Collectively, these observations indicated that exosomes are an important participant in the HO-ADSC-mediated promotion of EOC cell proliferation, migration, and invasion.

### 3.4. FOXM1 Expression Increased following Treatment with HO-ADSC Exosomes

**Figure 4:**
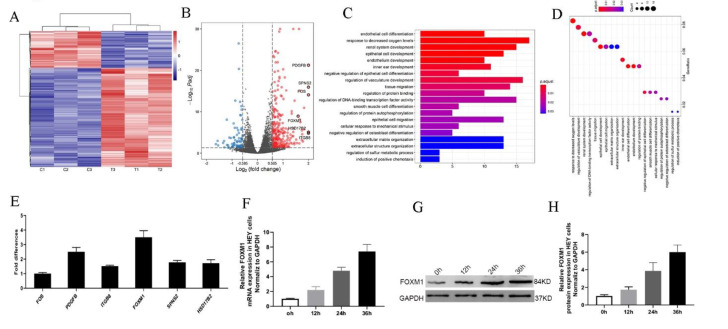
Transcriptome analysis of HEY cells treated with HO-ADSC exosomes. (**A**). Heatmap showing differentially expressed genes in HO-ADSC exosome-treated HEY cells and HEY control cells. (**B**). Volcano map showing the upregulated and downregulated genes from HO-ADSC exosome-treated HEY cells and HEY control cells. (**C**,**D**). Bioinformatics analysis was performed to determine clusters of differentially expressed genes with enriched molecular functions. (**E**). qRT–PCR experiments were performed to detect the expression of the top 6 upregulated mRNAs. (**F**). FOXM1 mRNA expression was measured by qRTPCR after treatment with HO-ADSC exosomes for 0, 12, 24, and 36 h. (**G**). Western blotting was performed to evaluate FOXM1 expression after treatment with HO-ADSC exosomes for 0, 12, 24, and 36 h. (**H**). Quantitative analysis of FOXM1 protein levels in HEY cells.

**Figure 5:**
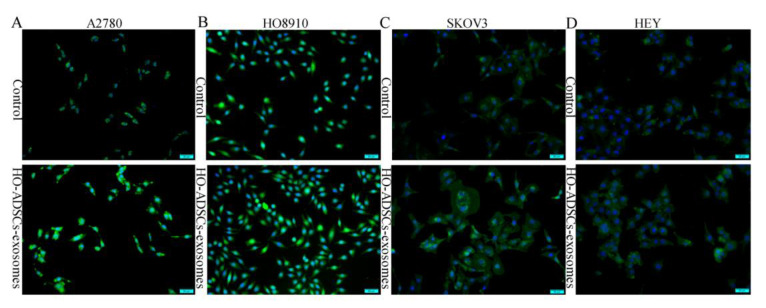
FOXM1 fluorescence expression in human EOC cells after treatment with HO-ADSC exosomes. Cell immunofluorescence was performed to evaluate the expression of FOXM1 (green) in EOC cells, and nuclei were stained with DAPI (blue). (**A**). Representative image of FOXM1 staining in A2780 cells in vitro. (**B**). Representative image of FOXM1 staining in HO8910 cells in vitro. (**C**). Representative image of FOXM1 staining in SKOV3 cells in vitro. (**D**). Representative image of FOXM1 staining in HEY cells in vitro.

To obtain a better understanding of how HO-ADSC exosomes regulate ovarian cancer cell proliferation, migration, and invasion, we analyzed RNA transcripts using RNA sequencing (RNA-seq) analyses in three pairs of EOC cell samples with or without HO-ADSC-exosome treatment. Heatmap analysis demonstrated that HO-ADSC-exosome treatment resulted in different mRNA expression levels (Figure 4A). A total of 261 mRNA candidates were differentially expressed between the HO-ADSC exosomes and control groups. Of these, 190 genes were upregulated, whereas 71 were decreased based on a fold change of 1.5 and *p* < 0.05. The variation in mRNA expression was observed in the volcano plot (Figure 4B). The predicted targets of the differentially expressed mRNAs were analyzed based on their Gene Ontology (GO) categories and pathway annotations, and we found that the differentially expressed genes were significantly associated with tissue migration and vasculature development (Figure 4C,D). We further confirmed the RNA-seq results of the top 6 upregulated mRNAs (FOS, PDGFB, ITGB8, HSD17B2, FOXM1, and SPNS2) by independent RT–PCR experiments in EOC cell lines. Interestingly, we found that only FOXM1 expression significantly increased in response to HO-ADSC-exosome treatment (Figure 4E). Consistent with this finding, we also verified that HO-ADSC-exosome treatment increased FOXM1 mRNA and protein expression in a time-dependent manner (0 h, 12 h, 24 h, and 36 h), as determined by qRT–PCR Western blot analysis (Figure 4F–H). Cell immunofluorescence was performed to evaluate FOXM1 expression in EOC cell lines (A2780, HO8910, SKOV3, and HEY) after HO-ADSC-exosome treatment. As shown in Figure 5A–D, an obvious increase in the FOXM1 immunofluorescence intensity was noted in HO-ADSC exosome-treated cells compared to control cells. Together, these data suggested that FOXM1 may play a role in HO-ADSC-exosome-mediated cell functional enhancement. Therefore, we selected FOXM1 for further investigation.

### 3.5. FOXM1 Knockdown Decreased HO-ADSC-Exosome-Mediated Promotion of EOC Cell Proliferation, Migration, and Invasion In Vitro 

**Figure 6:**
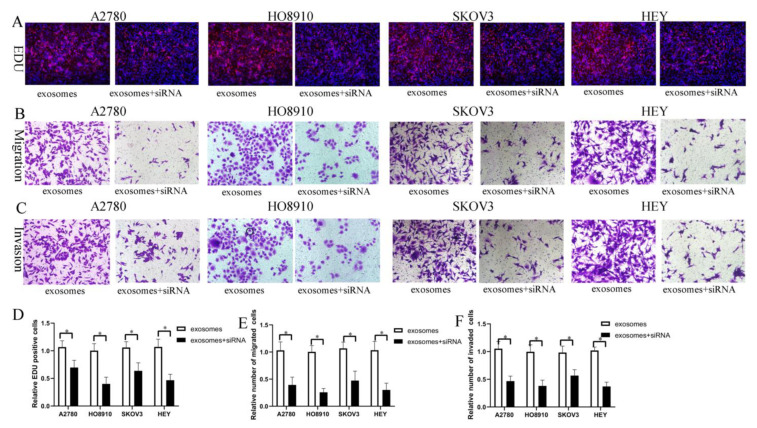
FOXM1 knockdown decreased HO-ADSC-exosome-mediated promotion of EOC cell proliferation, migration, and invasion in vitro. (**A**). The influence of FOXM1 silencing on human EOC cell proliferation was measured by EdU assay. (**B**). Influence of FOXM1 silencing on human EOC cell migration was measured by Transwell assay. (**C**). Influence of FOXM1 silencing on human EOC cell invasion was measured by Transwell assay. (**D**). Quantification of proliferation rates showed that the proliferative capacity of human EOC cell lines was significantly decreased after FOXM1 silencing. (**E**). Quantification of the migrated cells showed that human EOC cell migratory capacity was significantly decreased after FOXM1 silencing. (**F**). Quantification of the invasion rates of human EOC cell lines showed that the invasive capacity of human EOC cell lines was significantly decreased after FOXM1 silencing. * *p* < 0.05, n = 3.

It is widely accepted that FOXM1 is a typical proliferation-associated transcription factor involved in organismal development and tumorigenesis. Recent reports and our previous studies have shown that FOXM1 is an oncogene that plays a role in ovarian cancer growth and metastasis. To determine the impact of FOXM1 on the HO-ADSC-exosome-mediated promotion of EOC proliferation, migration, and invasion in vitro, EOC cell lines were transfected with FOXM1 siRNA and exposed to HO-ADSC exosomes. EdU assays showed that the increased proliferation of A2780, HO8910, SKOV3, and HEY cells following treatment with HO-ADSC exosomes was abrogated by FOXM1 siRNA (Figure 6A–F). Transwell migration and invasion assays indicated that the increased migratory and invasive abilities of the cells following treatment with HO-ADSC exosomes were reversed by FOXM1 siRNA. These results showed that HO-ADSC-exosome-mediated proliferation, invasion, and metastasis of EOC cells was associated with FOXM1 activation and that FOXM1 played a critical role in HO-ADSC-exosome-mediated promotion of EOC cell proliferation, migration, and invasion.

### 3.6. FOXM1 Regulated HO-ADSC-Exosome-Mediated EOC Progression by Targeting Cyclin F and KIF20A 

**Figure 7:**
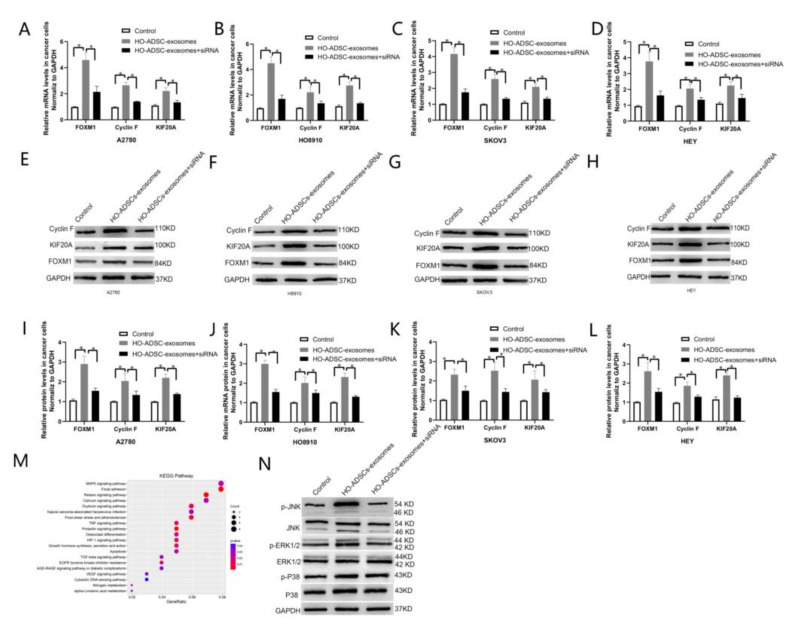
FOXM1 regulated HO-ADSC-exosome-mediated EOC progression by targeting Cyclin F and KIF20A. (**A**–**D**). FOXM1, Cyclin F, and KIF20A mRNA expression in A2780, HO8910, SKOV3, and HEY cells was determined by qRT–PCR. (**E**–**H**). FOXM1, Cyclin F, and KIF20A protein expression in A2780, HO8910, SKOV3, and HEY cells were measured using Western blot assays after FOXM1 knockdown. (**I**–**L**). Densitometric quantification of FOXM1, Cyclin F, and KIF20A expression in A2780, HO8910, SKOV3, and HEY cells after FOXM1 knockdown. (**M**). KEGG pathway enrichment based on the differentially expressed genes after HO-ADSC exosome treatment. (**N**). Western blotting was performed to evaluate MAPK signaling pathways. * *p* < 0.05, n = 3.

Although many downstream genes driven by FOXM1 have been elucidated, most of the downstream targets of FOXM1 in ovarian cancer remain unclear. Based on the integrative analysis of RNA-seq and ChIP-seq data in a recent report by our laboratory, we elucidated that FOXM1 regulates Cyclin F and KIF20A at the transcriptional level by directly binding to the promoter regions of these two genes [24]. Our laboratory also demonstrated that Cyclin F and KIF20A are involved in FOXM1-mediated ovarian cancer cell proliferation and metastasis, and high expression of Cyclin Fand KIF20A was associated with poor prognosis in patients with ovarian cancer [24]. These findings led us to focus on Cyclin F and KIF20A to explore the underlying molecular mechanisms involved in HO-ADSC-exosome-mediated proliferation, migration, and invasion of EOC cells. RT–PCR experiments were then performed in the A2780, HO-8910, SKOV3, and HEY cell lines, and the results showed that the mRNA expression of Cyclin F and KIF20A was significantly increased in HO-ADSC exosome-treated cells compared to control cells, and knockdown of FOXM1 significantly reduced the mRNA expression of Cyclin F and KIF20A induced by HO-ADSC exosomes (Figure 7A–D). Based on the above results, we also further performed Western blot analysis to detect the protein levels of Cyclin F and KIF20A, and we obtained results similar to those expected (Figure 7I–L). These data suggest that FOXM1 exerts its role partially by transcriptionally regulating Cyclin F and KIF20A.

### 3.7. FOXM1 Regulated HO-ADSC-Exosome-Mediated EOC Progression by Activating the MAPK Signalling Pathway

Given that KEGG pathway enrichment analysis suggested that the MAPK signaling pathway was one of the most enriched pathways in HO-ADSC-exosome-mediated EOC progression (Figure 7M), we studied whether MAPK signaling pathways, including the ERK1/2, *p* 38 and JNK pathways, were activated after HO-ADSC-exosome treatment. Western blot assays showed that p-ERK1/2 and *p*-JNK expression was strongly activated after treatment with HO-ADSC exosomes. FOXM1 knockdown reversed the impact of HO-ADSC exosomes on ERK1/2 and JNK phosphorylation in EOC cells. However, HO-ADSC-exosome treatment had no effect on the total levels of ERK1/2, *p* 38, and JNK or *p* 38 phosphorylation in EOC cells (Figure 7N). Collectively, these results demonstrate that the underlying mechanism by which HO-ADSC-derived exosomes modulate EOC cell functions is associated with ERK1/2 and JNK-MAPK signaling pathway activation.

### 3.8. HO-ADSC Exosomes Promoted EOC Tumorigenesis and Progression In Vivo

**Figure 8:**
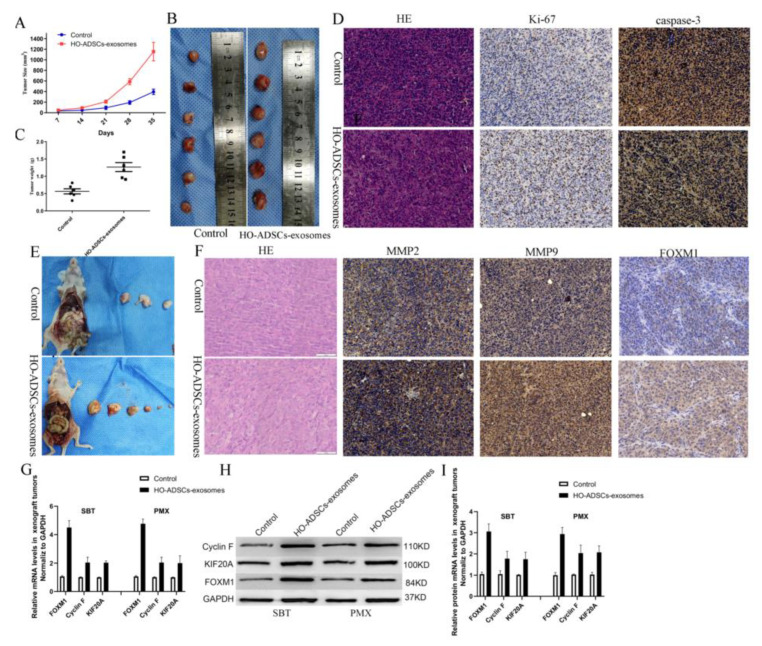
HO-ADSC exosomes promoted EOC tumorigenesis and progression in vivo. (**A**). The tumor growth curve of subcutaneous ovarian xenografts in mice. (**B**). Representative images of subcutaneous ovarian xenografts in mice. (**C**). Weight of subcutaneous ovarian xenografts in mice. (**D**). Representative HE staining and IHC staining for Ki-67 and caspase-3 in subcutaneous xenograft tumor tissues. (**E**). Representative images of peritoneal tumor nodules in mice. (**F**). Representative HE and IHC staining for MMP2, MMP9, and FOXM1 in peritoneal metastatic xenograft tumor tissues. (**G**–**I**). Cyclin F and KIF20A expression in peritoneal metastatic xenograft tumor tissues was determined by Western blot and qRT–PCR analysis. * *p* < 0.05, n = 3.

To further validate the functional role of HO-ADSC exosomes in ovarian cancer in vivo, we performed both subcutaneous tumorigenesis (SBT) and peritoneal metastatic xenograft (PMX) studies. The xenograft tumors were injected with HO-ADSC exosomes and control PBS in nude mice. As the subcutaneous xenograft assays showed, compared with the control groups, the HO-ADSC exosomes remarkably promoted the formation of subcutaneous tumors, thereby increasing the tumor volume and weight of the subcutaneous xenografts in the nude mice (Figure 8A–C). Moreover, we found that treatment with HO-ADSC exosomes increased the Ki-67 signal but decreased caspase-3 activity (cleaved caspase-3) compared to the control, as determined by immunohistochemistry (Figure 8D). In a peritoneal metastatic xenograft model, we found that injection of HO-ADSC exosomes substantially increased the number of metastatic nodules in the abdominal cavity (Figure 8E). We also evaluated the effect of HO-ADSC exosomes on MMPs and FOXM1 expression in tumor xenografts using immunohistochemistry. The results showed that the positive staining intensities of MMP2, MMP9, and FOXM1 were upregulated by treatment with HO-ADSC exosomes (Figure 8F). Moreover, we discovered that the Cyclin F and KIF20A mRNAs and proteins, which are involved in FOXM1 signaling, were also increased in the HO-ADSC exosome group compared with the control group. This finding was consistent with our in vitro results (Figure 8G–I). In conclusion, HO-ADSC exosomes promote the growth and metastasis of ovarian cancer cells in vivo by regulating FOXM1 signaling.

### 3.9. HOADSC-Exosomes Are Potentially Secreted into Ascites and Involved in Ovarian Cancer Growth and Metastasis 

**Figure 9:**
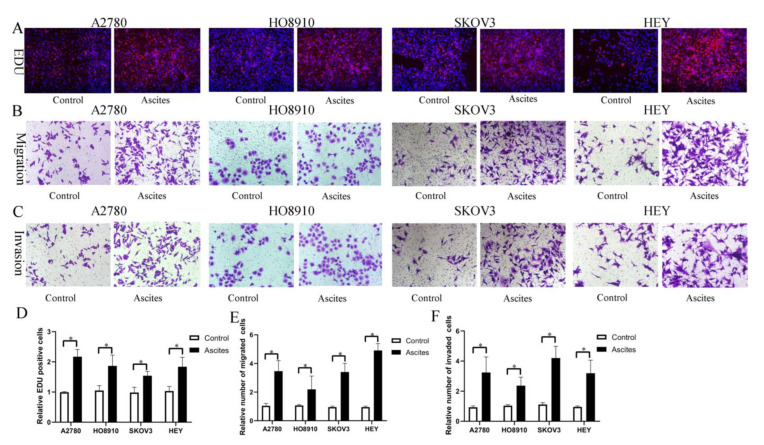
Ovarian cancer ascites increase human EOC cell proliferation, migration, and invasion (A2780, HO8910, SKOV3, and HEY) in vitro. (**A**). The effect of ascites on human EOC cell line proliferation was measured by EdU assay. (**B**). The effect of ascites on human EOC cell migration was measured by Transwell assay. (**C**). The effect of ascites on human EOC cell invasion was measured by Transwell assay. (**D**). Quantification of proliferation rates showed that the proliferative capacity of human EOC cell lines was significantly improved after ascites treatment. (**E**). Quantification of the migrated cells showed that human EOC cell migratory capacity was significantly improved after ascites treatment. (**F**). Quantification of the invasion rates of human EOC cell lines showed that the invasive capacity of human EOC cell lines was significantly improved after ascites treatment. * *p* < 0.05, n = 3.

Based on our findings in vitro and in vivo, it is reasonable to hypothesize that HO-ADSC exosomes may be secreted into ascites and possibly involved in ovarian cancer growth and metastasis. To examine the effects of malignant ascites on ovarian cancer cell growth and metastasis, ovarian cancer cell lines (A2780, HO-8910, SKOV3, and HEY) were treated with an acellular fraction of ascites isolated at the time of surgery from patients with high-grade serous ovarian carcinoma (HGSOC). The EDU assay showed that treatment with ascites for 3 days significantly promoted proliferation in ovarian cancer cell lines (Figure 9A,D). Transwell migration and invasion assays indicated that the migratory and invasive abilities of the cells were increased following treatment with ascites (Figure 9B,C,E,F). Therefore, these findings indicated that HO-ADSC exosomes may be secreted into ascites and may be involved in ovarian cancer growth and metastasis, at least in part.

## 4. Discussion

**Figure 10:**
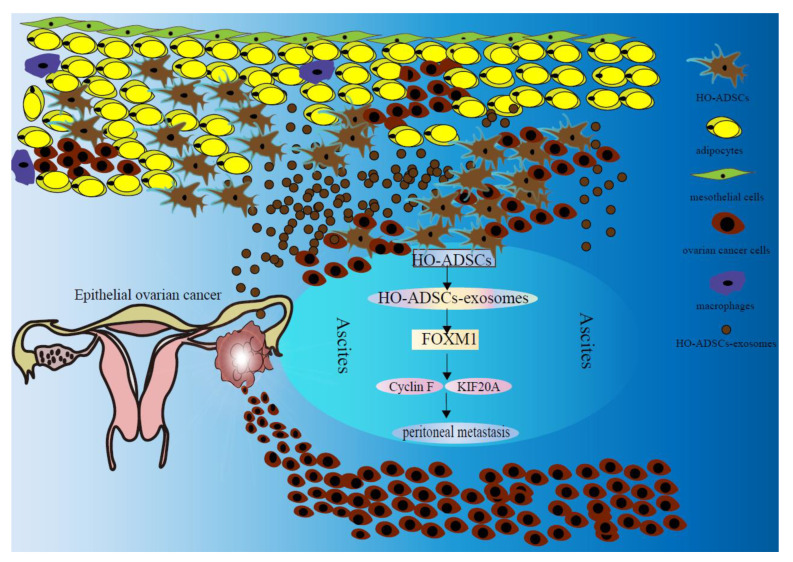
Schematic diagram shows the mechanism underlying HO-ADSC exosomes being secreted into ascites and their role in ovarian cancer growth and metastasis.

Ovarian cancer is the seventh most frequent malignancy worldwide, and the mortality rate ranks first among gynecological malignancies. The pathological types of ovarian cancer vary, of which EOC accounts for 85–90 percent of ovarian malignancies [25]. Despite significant advances in the treatment of ovarian cancer, the survival rate of EOC has not improved significantly in recent years [26]. It is generally known that EOC patients frequently develop implantation metastasis to the omentum and peritoneal organs and are commonly diagnosed at the late stage. However, the molecular basis underlying this tendency to seed the peritoneum is ambiguous [27]. In the present study, our goal was to investigate the role of HO-ADSCs in EOC metastasis and the underlying mechanisms. We found that HO-ADSCs increase the growth, migratory and invasive capabilities of ovarian cancer cells through an exosome-mediated mechanism. In addition, we demonstrated that HO-ADSC exosomes promote the growth and metastasis of ovarian cancer cells via a mechanism dependent on the FOXM1, Cyclin F KIF20A, and MAPK signaling pathways (Figure 10).

Increasing evidence supports that ADSCs can regulate the growth and metastasis of various malignant tumors, such as bladder cancer, cervical cancer, and ovarian cancer [28,29,30]. Yijing Chu et al. illustrated that ADSCs directly or indirectly cocultured with ovarian cancer cells accelerated ovarian cancer cell proliferation, invasion, and migration [11,13]. In contrast, another study by C. Khalil provided convincing evidence that ADSCs appear to inhibit the aggressiveness of ovarian cancer [31]. Thus, there are contradictory reports about the role of ADSCs in ovarian cancer development. There are many types of human EOC cell lines, such as SKOV3, CAOV3, OVCAR3, IGROV-1, CAOV-3, A2780, and IGROV-1. A2780, HO8910, SKOV3, and HEY cells are used in this study because they are typical EOC cell lines used in our laboratory. In our study, we first established that the human omentum plays a stimulatory role in promoting ovarian cancer growth and metastasis. Using ADSCs in the omentum, we further demonstrated that HO-ADSCs could increase the growth, migratory and invasive capabilities of ovarian cancer cells. These results indicated that HO-ADSCs promote EOC progression and metastasis through a paracrine mechanism. The differences in ADSCs in tumor progression reported by different groups might be due to their activation status [32].

Over the last decade, exosomes have been regarded as the newest information carrier of intercellular communication. Exosomes package a variety of protein components and nucleic acid molecules and then change the phenotype and function of recipient cells. At present, exosomes are considered to be the key messengers of intercellular communication and participate in various processes in tumor development, including angiogenesis, metastasis, drug resistance, and immune escape [33]. In this study, in vivo experiments indicated that exosomes are an important participant in HO-ADSC-mediated promotion of EOC cell proliferation, migration, and invasion. Using a nude mouse tumorigenesis model, we also verified that HO-ADSC exosomes promoted the tumorigenesis and progression of EOC by regulating FOXM1 signaling in vivo. However, more importantly, considering that exosomes can also be delivered to various body fluids, we further provided fresh evidence that HO-ADSC exosomes may be secreted into ascites and are potentially involved in ovarian cancer growth and metastasis. This explains why patients with malignant ovarian tumors are often complicated with ascites and frequently develop peritoneal dissemination, and this is the significance of our study. In addition, we speculate that targeting the HO-ADSC exosomes may be a potential approach to treat advanced ovarian cancer with omentum metastasis.

To understand the mechanism by which HO-ADSC exosomes increased proliferation, migration, and invasion in ovarian cancer cell lines, we focused on FOXM1, as previous investigations have shown that the FOXM1 pathway is commonly activated in various solid cancers [34]. Recently, reports by our laboratory have demonstrated that FOXM1 is an oncogene implicated in the growth and metastasis of ovarian cancer [24]. Moreover, our data support that Cyclin F and KIF20A are involved in FOXM1-mediated ovarian cancer formation and development, and high Cyclin F and KIF20A expression was associated with poor prognosis in patients with ovarian cancer [20]. Consistent with the findings from a previous study of our laboratory, we provided new evidence that FOXM1 played a critical role in HO-ADSC-exosome-mediated ovarian cancer growth and metastatic progression, and FOXM1 exerts its role partially by transcriptionally regulating Cyclin F and KIF20A expression. MAPK signaling pathways are one of the most important signaling pathways that are closely related to cancer cell growth and metastasis. In addition, based on the results of the KEGG pathway enrichment analysis, we also uncovered the underlying mechanism by which HO-ADSC exosome-modulated EOC cell functions were associated with ERK1/2 and P38 MAPK signaling pathway activation. The results were generally consistent with a past study showing that exosomes derived from human bone marrow mesenchymal stem cells contributed to tumor progression through the ERK1/2 and p38 MAPK pathways [35].

Despite promising findings, our study has some limitations. In this study, we focused on confirming the effects of HO-ADSC exosomes on EOC metastasis and the related mechanism. However, the specific key factor contained in HO-ADSC exosomes that modulates FOXM1 signaling pathways is unclear. Based on existing information, the possible regulation of FOXM1 may be active substances such as proteins, RNA, and lipids encapsulated in exosomes. In future studies, we will assess the expression patterns in HO-ADSC exosomes. In addition, in vivo animal experiments were performed using subcutaneous tumorigenesis and peritoneal metastatic xenograft models in nude mice. However, the anatomic and histological structures of the mouse and human omentum are not identical, and more clinical studies are needed.

## 5. Conclusions

Collectively, our data indicated that HO-ADSCs enhance the migration, invasion, and proliferation of ovarian cancer cells through exosome-mediated FOXM1 mechanisms and that HO-ADSC exosomes could be secreted into ascites and exert a tumor-promoting effect in EOC peritoneal metastasis. These findings provide new insights into understanding the clinical phenomenon that EOC patients are often accompanied by ascites and frequently spread to the visceral adipose tissue of the omentum. With this knowledge, novel therapeutic strategies and other clinical interventions underlying metastasis may also be explored for EOC patients.

## Data Availability

The data that support the findings of this study are available from the corresponding author upon reasonable request.

## Supplementary Materials

The following supporting information can be downloaded at: https://www.mdpi.com/article/10.3390/cells11213392/s1, Figure S1: A. Morphological observation of HO-ADSCs. B. Adipogenic differentiation of HO-ADSCs detected by Oil Red O staining. C. Osteogenic differentiation of HO-ADSCs detected by Alizarin Red staining. D. Chondrogenic differentiation of HO-ADSCs detected by Alcian Blue staining. Figure S2: Flow cytometric analysis of HO-ADSC-positive markers and -negative markers. A. CD19; B. CD19; C. CD34; D. CD45; E. CD73; F. CD90; G. CD105; H. HLA-DR. Figure S3: A. HO-ADSC-exosome morphology observed by TEM. B. Western blot analysis of exosome surface markers (TSG101, CD63 and HSP70). Figure S4: PKH26-labelled HO-ADSC exosomes were cultured with EOC cells. Fluorescence images showed DAPI-stained EOC cell nuclei (A) and PKH26-labelled exosomes (B). The merged picture shows the cellular internalization of HO-ADSC exosomes into EOC cells (C).

## Abbreviations

MSC: Mesenchymal stem cell; EOC: Epithelial ovarian cancer; ADSCs: Adipose-derived mesenchymal stem cells; HO-ADSC: Human omental Adipose-derived mesenchymal stem cell; HO-CM: Human omental tissue conditioned medium; exosomes secreted by HO-ADSCs: HO-ADSC exosomes; IHC: Immunohistochemistry; qRT-PCR: Quantitative real-time PCR; SBT: Subcutaneous tumorigenesis; PMX: Peritoneal metastatic xenograft; TEM: Transmission electron microscopy; HE: Hematoxylin and eosin.
