# Supplementary material for: Exosomes from Human Omental Adipose-Derived Mesenchymal Stem Cells Secreted into Ascites Promote Peritoneal Metastasis of Epithelial Ovarian Cancer
Source: Cells. 2022 Oct 27;11(21):3392. doi: 10.3390/cells11213392 (PMC9655202; doi:10.3390/cells11213392)
Supplement: Supplementary file 1 [file cells-11-03392-s001.zip › cells-1937169-supplementary.pdf]

Supplemental materials

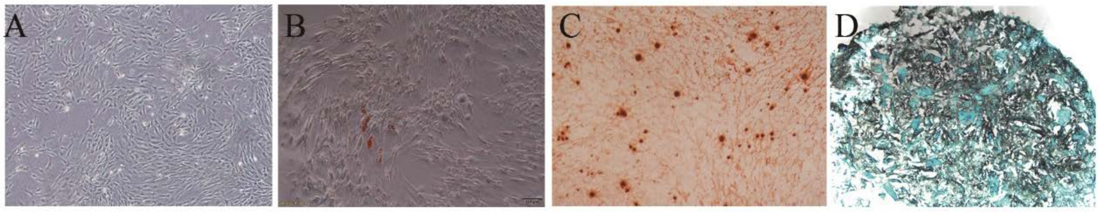

Figure S1. A. Morphological observation of HO-ADSCs. B. Adipogenic differentiation of HO-ADSCs detected by Oil Red O staining. C. Osteogenic differentiation of HO-ADSCs detected by Alizarin Red staining. D. Chondrogenic differentiation of HO-ADSCs detected by Alcian Blue staining.

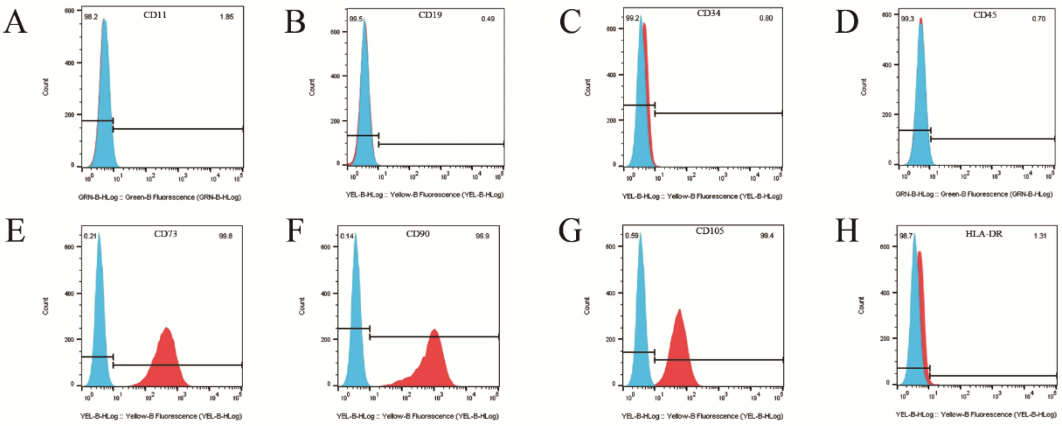

Figure S2. Flow cytometric analysis of HO-ADSC-positive markers and -negative markers. A. CD19; B. CD19; C. CD34; D. CD45; E. CD73; F. CD90; G. CD105; H. HLA-DR.

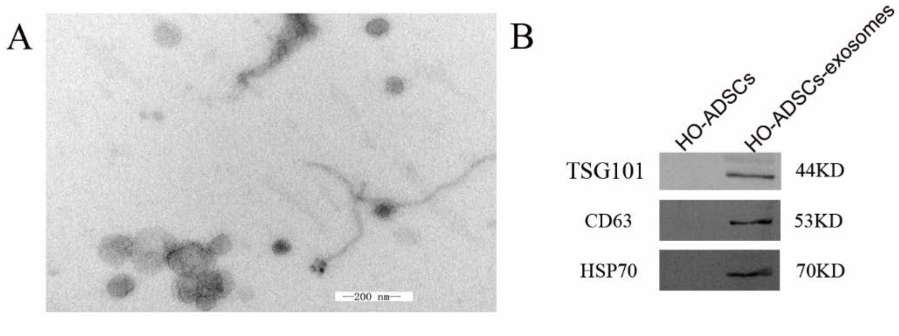

Figure S3. A. HO-ADSC-exosome morphology observed by TEM. B. Western blot analysis of exosome surface markers (TSG101, CD63 and HSP70).

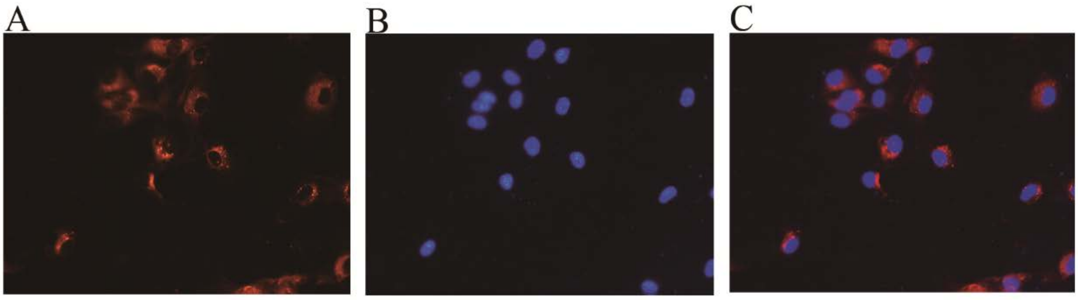

Figure S4. PKH26-labelled HO-ADSC exosomes were cultured with EOC cells. Fluorescence images showed DAPI-stained EOC cell nuclei (A) and PKH26-labelled exosomes (B). The merged picture shows the cellular internalization of HO-ADSC exosomes into EOC cells (C).
